# Graves’ disease and papillary thyroid carcinoma: case report and literature review of a single academic center

**DOI:** 10.1186/s12902-022-01116-1

**Published:** 2022-08-09

**Authors:** Marilyn A. Arosemena, Nicole A. Cipriani, Alexandra M. Dumitrescu

**Affiliations:** 1 Department of Adult and Pediatric Endocrinology, Diabetes and Metabolism, The University of Chicago, 5758 S. Maryland Ave, Chicago, IL 60637 USA; 2 Department of Pathology, The University of Chicago, 5841 S. Maryland Ave, Chicago, IL 60637 USA

**Keywords:** Graves’ disease, Thyroid cancer, Autoimmunity, Thyroidectomy, Case report

## Abstract

**Background:**

Graves’ disease (GD) and papillary thyroid cancer (PTC) can be concomitant. The existence of a link between these entities has long been investigated, but a clear correlation hasn’t been established. We report a case of GD resistant to medical treatment in which surgery revealed unsuspected PTC and we aim to study the prevalence of PTC in Graves’ disease, its clinical characteristics and review of the literature.

**Case presentation:**

Report of a 32 yo man who presented with weight loss and was found to be biochemically hyperthyroid. Antibodies were positive. Incremental doses of methimazole provided no improvement in thyroid tests. Hypervascularity and a spongiform nodule were noted on ultrasound. Thyroid uptake and scan showed 70.2% uptake. Thyroidectomy was performed due to inadequate therapeutic response. Pathology revealed PTC with extrathyroidal extension and positive lymph nodes. A retrospective review (2000–2021) and literature review of PTC in GD was performed. Clinical data were reviewed. Statistical analysis was calculated to identify correlations. 243 GD patients had total thyroidectomy at an academic center, 50 (20%) had PTC, 14% were microcarcinomas. 76% of cases were less than 55yo, 82% female, 78% stage 1, PTC diagnosis was incidental in 48%, hyperthyroidism was difficult to treat in 10% and only 2% had recurrence of PTC. There was no correlation between demographic or clinical data.

**Conclusions:**

Evidence is controversial with some studies showing GD does not affect PTC prognosis. PTC may not be well recognized in GD, pre-operative assessment should consider risk of cancer.

## Background

Graves’ disease (GD) is the most common cause of hyperthyroidism. It is an autoimmune condition in which activating antibodies, thyroid stimulating immunoglobulin (TSI), induce thyroid hormone overproduction. The manifestations depend on the age of the patient as well as the severity and the duration of hyperthyroidism. Spontaneous remission occurs in a small proportion of patients. The most common used therapies include antithyroid drugs, radioactive iodine (RAI) or thyroidectomy [1].

Papillary thyroid carcinoma (PTC) is the most common endocrine malignant neoplasm worldwide with an increasing record number of new cases every year. It represents the 8th most diagnosed cancer worldwide [2, 3].

Some studies have shown that thyroid autoimmunity and PTC can be concomitant, however a pathogenic correlation has not been established. A link between inflammation and carcinogenesis is well known since 1863 when Virchow showed leukocytes in cancer tissues and suggested an association with the development of cancer [2].

Additional evidence suggests that autoimmunity and inflammation are risk factors for PTC. It is hypothesized that the tumor microenvironment and inflammatory cells are connected to fibroblasts, endothelial cells, and extracellular matrix through cytokines, chemokines, and adipocytokines [2]. Hashimoto’s thyroiditis and GD patients show expression of a biomarker of oxidative DNA damage, which possibly favors development of PTC [3].

The incidence of PTC has increased worldwide and most recently reported to be 2–17% [4]. Malignancy rate of palpable thyroid nodules in GD patients has reported to be as low as 2.3% to up to 45.8% [5]. The etiology of the increased risk of PTC in GD is not completely understood but TSI may play a role [6, 7].

It continues to be controversial if GD is a risk factor for PTC and if patients with GD and PTC have worse prognosis. The objective of our study is to report a case of GD resistant to medical treatment in which surgery revealed unsuspected PTC, to review the prevalence, clinical characteristics and prognosis of PTC in GD at our academic center and perform a literature review of this interaction.

## Case presentation

A 32 year old Hispanic male with no known past medical history presented to his primary care physician with 20 lbs weight loss, without other symptoms. Vital signs revealed blood pressure of 133/76 mm Hg, heart rate of 80 beats per minute, respiratory rate of 17 respirations per minute and oxygen saturation of 98%. Physical exam revealed a thin man in no distress, head and neck exam revealed no orbitopathy with a thyroid gland that was not enlarged, cardiovascular exam revealed a regular rate and rhythm, lungs were clear to auscultation bilaterally, no abdominal tenderness, neurologic exam showed no tremors or hyperreflexia.

**Fig. 1:**
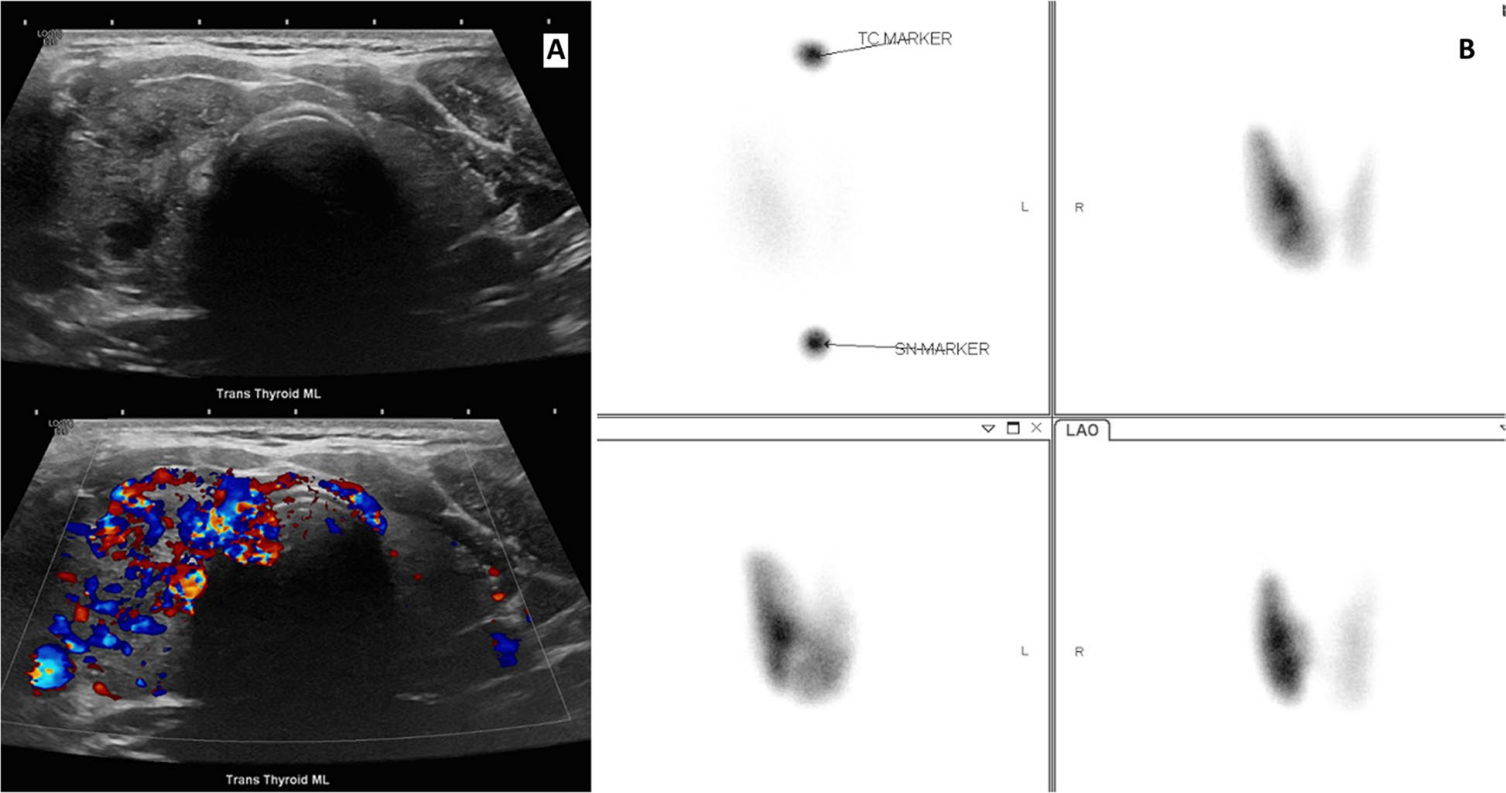
**A** Thyroid Ultrasound performed at the time of diagnosis. **B** Thyroid uptake and scan

Laboratory results revealed normal complete blood cell count and basic metabolic panel and TSH of < 0.01 mcU/mL that prompted referral to Endocrinology. He denied palpitations, hyperdefecation, tremors, skin changes or anxiety. Further workup revealed a free T4 (fT4) of 3.73 ng/dL and total T3 (TT3) of 291 ng/dL. Methimazole (MMI) 20 mg BID and propranolol 20 mg BID were started. Ultrasound (US) revealed a hypervascular gland with an asymmetric ill-defined hypoechoic enlarged right to mid lower pole nodule measuring 1.2 × 1.6 × 1.5 cm indicative of subacute thyroiditis (Fig. 1A).

Thyroid uptake and scan revealed uniform enlargement of the right greater than the left thyroid gland, no cold nodules were noted. The 4 h-RAI uptake was 47.2% and the 24-h was 70.2% (Fig. 1B). Thyroid function tests on MMI were monitored. One week later, fT4 increased to 4.75 ng/dL and TT3 increased to 307 ng/dL with TSI of 8, thyroglobulin of 53 ng/mL and negative thyroglobulin antibody and TPO antibody.

MMI was increased to 20 mg TID and repeat labs in one week showed persistently elevated fT4 of 4.65 ng/dL and TT3 of 290 ng/dL. Prednisone 20 mg was added for suspected component of thyroiditis and one week later fT4 decreased to 3.82 ng/dL and TT3 decreased to 230 ng/dL. This medication regimen was administered for two weeks, however thyroid hormone levels remained elevated fT4 2.73 ng/dL and TT3 202 ng/dL at which time prednisone was discontinued as the inflammatory markers (Erythrocyte sedimentation rate and C-reactive protein) were negative. To address this apparent resistance to MMI, cholestyramine 4 g TID was added for one week with no improvement in thyroid tests, fT4 was 2.54 ng/dL and TT3 was 192 ng/dL. Of note, these thyroid hormone levels were measured after being for 2 months on MMI 60 mg daily.

Repeated thyroid US to follow up on the nodule revealed a heterogenous, hypervascular gland with a nodule in the right lobe and isthmus (1.7 × 3.3 × 1.1 cm), spongiform with solid areas and a borderline enlarged right level 2 lymph node with normal morphology. Considering the presence of the nodule and the apparent inefficiency of the anti-thyroid treatment to control the hyperthyroid hormone levels the patient was referred to Endocrine surgery. Due to the nodule having benign features on US (no calcifications, spongiform, absent ill-defined border, and no suspicious lymph node) and not being cold, fine needle aspiration (FNA) was not recommended. In preparation for surgery, he received potassium iodine (KI) for 10 days which resulted in fast normalization of thyroid hormone levels. Total thyroidectomy (TT) was performed with no complications.

Pathology revealed 1.5 cm PTC in the right lower lobe-isthmus with suspicion for extrathyroidal extension into fibroadipose tissue, follicular hyperplasia with dilated follicles, papillary like structures and interfollicular fibrosis consistent with GD, and metastatic PTC in 2 level VI lymph nodes (2/2) with the largest focus of 0.4 cm, no extranodal extension. Immunostaining for BRAF VE1 showed diffuse moderate cytoplasmic expression in tumor cells, which is predictive of BRAF V600E mutation. TSI remained elevated at 5.9 four weeks after surgery. The patient subsequently underwent lymph node mapping that revealed a left lateral level II lymph which measured 1.4 × 0.4 cm. FNA revealed no malignancy. Due to stage I PTC (presumed BRAF V600E mutated), 50 mCi RAI was given. Total body scan revealed three foci of functioning thyroid tissue and regional lymph node metastases. Currently he is doing well awaiting his 6-month follow up scan.

Following approval of the Institutional Review Board, a retrospective chart review of patients with GD was performed. Selected patients were seen at our institution between January 2000 and April 2021. GD was confirmed by either positive antibodies and clinical history of hyperthyroidism or a compatible diagnosis based on thyroid uptake and scan results. If present, PTC was confirmed based on pathology report. Fifty patients met criteria for both diagnoses. Demographic and clinical data was collected. Informed consent was waived as the study was retrospective in nature.

From January 2000 to April 2021, 243 patients with GD underwent TT. Out of the 243, 50 patients (20%) had a diagnosis of both GD and PTC. Clinical and demographic characteristics revealed that 76% patients were < 55 years of age with a mean age at diagnosis of 44.5 years, 82% were female and 46% had positive TSI. 10% of the cases had difficult to treat hyperthyroidism, meaning not responding to average doses of thionamides (Table 1).

**Table 1:** Demographic and clinical characteristics of patients with Graves’ disease and PTC

Demographic/clinical data	*n* = 50	%
**Age**		
< 55	38	76%
> = 55	12	24%
**Sex**		
Female	41	82%
Male	9	18%
**TSI**		
Positive	23	46%
Negative	2	4%
Unknown	25	50%
**Initial stage**		
Stage 1	39	78%
Stage 2	11	22%
Stage 3	0	0%
Stage 4	0	0%
**Evolution**		
Recurrence	1	2%
No recurrence	21	42%
Unknown	28	56%
**Diagnosis**		
Incidental	24	48%
Suggested	26	52%
**Treatment used**		
Thyroidectomy	38	76%
Lobectomy	6	12%
Thyroidectomy + RAI	6	12%
**Hyperthyroidism difficult to treat?**^a^		
Yes	5	10%
No	45	90%
**Focality**		
Unifocal	33	66%
Multifocal	17	34%
**BRAF**		
Yes	7	14%
No	4	8%
Unknown	39	78%
**Size**		
Carcinoma (< = 1 cm)	36	72%
Carcinoma (> 1 cm)	14	28%

^a^Hyperthyroidism difficult to treat was defined as a Yes if documented by primary Endocrinologist that patient was not responding to average thionamide doses and needing to refer patient for definitive therapy due to absent response

*TSI* Thyroid stimulating antibody, *RAI* Radioactive iodine, *PTC* Papillary thyroid cancer

Pathology revealed that most cancers were unifocal (66%), gland had an average weight of 29 g. Out of the 50 cases, 11 samples were tested for BRAF VE1 immunostain and 63% were found to be positive. When looking at size, 72% of PTC were microcarcinomas (< = 1 cm). Based on the AJCC Staging, 78% were Stage I, 22% were Stage II and there were no cases with Stage III or IV. When looking at the evolution and prognosis, so far only 2% of PTC recurred.

## Discussion and conclusions

A casual correlation between GD and PTC continues to be controversial. In our study we present a case of GD that proved challenging due to a combination of features. GD presented as minimally symptomatic in a young male in which the only symptom was weight loss, in part intentional, without palpitations, tremors, or gastrointestinal symptoms, normal vitals signs and no goiter. One of the unusual features was the difficultly in treating the high levels of thyroid hormones as patient did not adequately respond to high doses of MMI (60 mg daily for 2 months). Poor compliance to treatment was initially considered however the patient was a family member of a physician who was able to confirm patient was taking medication as prescribed. There are 2 case reports about GD resistant to MMI, in one of them the patient was on 150 mg of MMI with no response and only responded to iodine, steroids and lithium [8]. Another case report described a patient with GD resistant to MMI (requiring 150 mg daily), in this case serum and intrathyroidal MMI concentrations were measured and intrathyroidal MMI concentration was low [9]. The underlying mechanisms of the resistance remain unclear. Hypotheses considered in these cases include malabsorption or liver disease affecting pharmacokinetics of MMI, large thyroid glands impairing uptake, or manifesting greater metabolism and excretion of MMI [8, 9].

The other unusual finding was that the diagnosis of PTC escaped detection on US with appearance initially as a subacute thyroiditis then as a spongiform nodule and the thyroid uptake and scan did not show a cold area in the respective location, possibly confounded by the superimposed increased uptake due to GD. As the patient was hyperthyroid and the imaging was reassuring, fine needle biopsy was not pursued. Incidental thyroid cancer has also been reported in prior retrospective studies. Keskin et al. reported 8% incidental thyroid cancer cases in patients with GD. The presence of nodules increases the chance of finding cancer, a metanalysis revealed that the presence of at least one nodule is associated with a fivefold increase in thyroid cancer risk [10]. The most common cancer reported in GD is PTC and microcarcinomas are particularly common [7, 11, 12].

The incidence of PTC in GD has been increasing overtime and the rate of cancer in GD is higher when compared to euthyroid controls [4, 6, 7, 13]. The possible mechanism behind remains to be elucidated, some studies report that antibodies may be the reason behind this association. As TSI stimulates autonomous signaling through the TSH receptor, a pathway that results in thyroid gland growth, the potential role of TSI as growth factor for PTC in setting of GD is being questioned. However, so far, studies have shown no significant difference between TSI titers and PTC development [14]. Interestingly a recent study revealed that higher TRab titers are associated with lower risk of malignancy [7].

There is persistent debate in the literature, with some studies showing GD affects the prognosis of PTC while other show that thyroid cancer in GD is not more aggressive than in euthyroid patients. A metanalysis from 2019 revealed that GD has a significantly higher risk of associated multifocality/multicentricity and distant metastasis at the time of cancer diagnosis but is not associated with PTC related mortality or recurrence [15]. Pellegriti et al., reviewed patients with differentiated thyroid cancer and GD and showed a higher disease-specific mortality rate compared to euthyroid group. GD was characterized by a high mortality, with elevated rates of metastasis and relapse [16]. Case control studies have also revealed that PTC associated with GD show aggressive behavior even when tumor characteristics are favorable [17]. On the contrary, some studies suggest that GD may be associated with a worse outcome of coexisting PTC only if cancer is ≥ 1 cm [11].

Reassuring data comes from a propensity scored matching study that evaluated > 3000 patients with TT and revealed that GD does not affect the prognosis of PTC and that thyroid cancer in patients with GD is not more aggressive [18]. Two other previous studies, one evaluating microcarcinomas in GD and another evaluating thyroid cancer in general revealed prognosis is similar to euthyroid patients [19, 20].

Evidence regarding PTC prognosis in GD is controversial with some studies showing GD does not affect PTC prognosis. This case report illustrates that PTC may not be well recognized in GD and that although prevalence is low, pre-operative assessment should consider risk of cancer as GD can obscure and confound the imaging when GD and thyroid nodules coexist. Our retrospective review revealed most patients had a low rate of recurrence and potentially favorable prognosis.

## Data Availability

Due to patient privacy we cannot share PHI. All retrospective data is shown in Table 1. We will be able to provide only de-identified data of our patients to confirm/reproduce analysis. These could be requested by sending an email to the corresponding author Dr. Arosemena.
